# Health and well-being of university students before and during COVID-19 pandemic: A gender comparison

**DOI:** 10.1371/journal.pone.0261346

**Published:** 2021-12-14

**Authors:** Sunna Gestsdottir, Thordis Gisladottir, Runa Stefansdottir, Erlingur Johannsson, Greta Jakobsdottir, Vaka Rognvaldsdottir

**Affiliations:** 1 Center of Sport and Health Sciences, School of Education, University of Iceland, Reykjavik, Iceland; 2 Department of Sport, Food and Natural Sciences, Western Norway University of Applied Sciences, Bergen, Norway; Medical University of Vienna, AUSTRIA

## Abstract

**Objective:**

COVID-19 has affected people’s health in various ways. University students are a particularly sensitive group for mental and physical health issues. The aim of this study was to assess and compare the mental and physical health of male and female first-year university students during and before COVID-19.

**Method:**

Total of 115 first-year university students (54% male) answered questions about mental and physical health. The students were asked to estimate their physical activity, sedentary behavior, loneliness, stress, and sleep quality during COVID-19 opposed to before the pandemic.

**Result:**

Males had fewer symptoms of anxiety and depression, and their self-esteem was higher than females (p<0.05). Over 50% of both genders estimated their mental health to be worse than before COVID-19. Larger proportion of males (69%) compared to females (38%) estimated that their physical health had worsened than before the pandemic. Larger proportion of females (38%) than males (14%) experience increased loneliness and stress (68% vs. 48%). Over 70% of both genders estimated increased sedentary behavior than before the pandemic, and larger proportion of males (76%), compared to females (56%), estimated that they were less physically active than before COVID-19. About 50% of participants estimated their sleep quality was worse than before COVID-19.

**Conclusion:**

University students estimated their mental and physical health to have deteriorated during the pandemic. Therefore, it is important that the school and healthcare systems assist students in unwinding these negative health and lifestyle changes that have accompanied the pandemic.

## Introduction

The coronavirus outbreak, COVID-19, which the World Health Organization declared a global pandemic in March 2020 [1], has upended people’s lives across the globe. To reduce the rapid spread and death caused by COVID-19, governments enforced comprehensive restrictions (physical/social distancing) and even lockdowns that limited the social gathering of people, including exercise. In any pandemic, it is natural for individuals to feel anxious and afraid. The social costs of an increased virtual existence concern health professionals, governments, and educational institutions.

Studies conducted during the pandemic indicate a downturn in adults’ mental and physical health, including an increase in suicidal ideation and substance use disorder [2]. Mental health of young individuals has declined [3], and their physical activity (PA) decreased [4], while sedentary behavior has increased [5], and sleep quality [6] worsened after the pandemic. Previous studies have reported that lifestyle choices and gender influence health outcomes and mortality later on [7]. Effects of COVID-19 are seen across populations, but university students are a particularly sensitive group for mental and physical health issues during a pandemic. They are already facing challenges following major life transitions during emerging adulthood, including academic, economic, and social responsibilities [8]. In addition, the unforeseen move of university education from face-to-face to online delivery has been challenging. Even before the pandemic, there were indications of declining mental health among emerging adults.

Over the past decade, there has been an increase in major depressive episodes [9], and more students seek treatment for anxiety and depression [10]. Studies regarding gender differences in mental health indicate that males have better body image, higher self-esteem, less stress, anxiety, and depression [11–13]. Results for gender differences on perceived loneliness among university students have been mixed [14]. Restrictions during the pandemic, such as suspension of schools and lockdowns, led to a dramatic decrease in social connection, educational engagement, and opportunity to exercise, which all protect against ill-being.

Recently, few studies have compared students’ mental health by gender during the COVID-19 pandemic. A France study conducted during the first containment found that females scored significantly higher on depression, anxiety, and distress measures compared to males [15]. However, Cao et al found no significant difference in gender when exploring anxiety among college students during the COVID-19 pandemic in China [16]. Further, Meda et al explored student´s mental health before, during and after the COVID-19 lockdown in Italy and reported that students experienced worse depressive symptoms during the lockdown, independent of gender [17]. Thus, results seem to vary depending on pandemic time and restriction but a recent systematic review and meta-analysis reporting students’ mental health status during the pandemic showed that in general females reported more depressive and anxiety symptoms compared with males [18].

Most studies evaluating mental and physical health status during COVID-19 lack information on health before COVID-19 and data regarding gender differences are scarce. Current study is one of the few to explore the effects of the COVID-19 pandemic on physical and mental well-being of first-year university students and one of the first that focuses on gender differences.

The aim of this study was to assess and compare the mental and physical health of male and female first-year university students during and before Covid-19.

## Methods

### Participants and data collection

**Fig 1:**
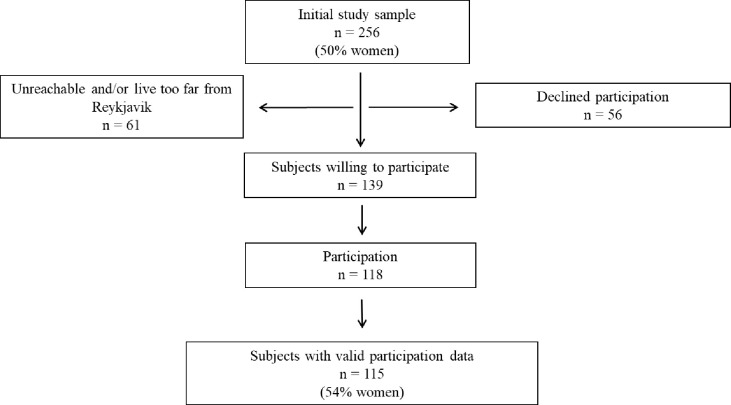
Study participation.

The study sample was randomized from all first-year students registered in a public university in Iceland during spring 2021. Subjects eligible for participation were young adults under 32 years of age. A total of 495 students, 128 males and 366 females, fit the participation criteria. For gender comparison, stratified randomization was used. All 128 male students were invited to participate, and 128 (of the 366) female students were randomly offered participation. The final study sample consisted of 256 students resulting in 118 participating, of which 63 were males, 54 were females, and one identified its gender as other than male or female. Three implausible or invalid subjects were removed from the final study sample (Fig 1). Non-participation (n = 138) was mainly due to absence from school during measurements, schedule conflicts, or lack of interest in the study, although 61 subjects did not reply to an e-mail or a phone call.

At the beginning of the 2021 spring semester (January), all selected participants were sent information regarding the study through their university e-mail. In addition, participants received a follow-up phone call, where the study procedure was explained and lab time scheduled. To minimize no-shows, participants received a reminder via text message two days before their appointment, and those who did not arrive were contacted. At the lab, participants answered a questionnaire on their cell phones (due to COVID-19 sanitary reasons). Those who did not have a smartphone or had any issues accessing the survey were loaned an iPad (four participants). During the time of measurements the university used mostly online teaching due to restrictions regarding all individuals needed to be two meters apart, wear a mask over face and mouth and no more than 50 people were allowed in the same room/space at a time. The vaccination against COVID-19 had barely started (only health professionals at hospitals and primary health care centers had received the vaccination in January 2021). No lockdown has been in Iceland due to COVID-19 only restrictions regarding social/physical distancing and how many people can gather.

Participants provided written consent, and study procedures were conducted according to the guidance provided in the Declaration of Helsinki. The National Bioethics Committee and the Icelandic Data Protection Authority approved the study (VSNb2020100026/03.01).

### Measures

#### Anxiety

Anxiety was assessed with the General Anxiety Disorder-7-item scale (GAD-7) [19]. GAD-7 is a seven-item questionnaire that assesses general anxiety by asking about symptoms for the previous two weeks. Answers for each question are on a four-item response scale. Cronbach’s alpha was used to evaluate internal consistency, α = 0.91.

#### Body image

Body image was evaluated with five questions from the Body and Self-Image subscale of the Offer Self-Image Questionnaire (OSIQ), which asks about general feelings about oneself [20]. Participants were asked how well they agreed with the statements about their appearance. All items were rated on a four-point response scale, where 1 = not at all true of me, and 4 = true of me. Higher scores reflect a better body image. Cronbach’s alpha was used to evaluate internal consistency, α = 0.71.

#### Depression

Depression was assessed with a 10-item depression sub-scale from the SCL-90, which asks about symptoms of depression the previous week [21]. It is scored on a four-point Likert scale rated from 1 (seldom) to 4 (often). An example of a question is, “Did you feel that the future is hopeless?”. Cronbach’s alpha was used to evaluate internal consistency, α = 0.89.

#### Self-esteem

Self-esteem was assessed with the widely used Rosenberg self-esteem scale [22], which asks about general feelings about oneself. The scale consists of ten statements, each rated as negative or positive, with four response options ranging from “strongly disagree” (1) to “strongly agree “(4). Cronbach’s alpha was used to evaluate internal consistency, α = 0.87.

#### Loneliness and stress

Perceived loneliness and stress during COVID-19 were estimated with two separate questions: “Have you experienced loneliness/stress due to the pandemic?” Response options were: I have experienced 1) much loneliness/stress, 2) average loneliness/stress, 3) little loneliness/stress, and 4) no loneliness/stress. A binary variable was created, combining response options 1 and 2 in one category and 3 and 4 into another.

#### Mental health, physical health, and sleep quality

An estimation of mental and physical health as well as the quality of sleep compared to before the pandemic was made with three questions: “How do you estimate your mental health/physical health/sleep quality compared to the time before COVID-19?” Response options were 1 = worse, 2 = the same, and 3 = better. Participant’s body composition was evaluated using self-reported height and weight with the following questions: “What is your body weight without clothes (in kilograms)?” and “What is your height (in centimeters)?” Body mass index (BMI) was calculated by dividing weight by height squared (kg/m^2^).

#### Physical activity and sedentary behavior

PA and sedentary behavior were assessed with the following two questions: “During COVID-19, how do you estimate your PA/sedentary behavior compared to the time before the outbreak of the pandemic?” Response options were 1 = less, 2 = equal, and 3 = more.

#### Demographics

Participants reported on their parents’ educational attainment, which was used as a proxy for socioeconomic status: “What education has your mother/father completed?” Response options were 1 = "Completed a university degree", 2 = "Started a university degree but did not complete", and 3 = "Completed secondary education", 4 = "Started secondary education but did not complete", 5 = "Completed compulsory education or less", and 6 = "Do not know".

### Statistical analyses

Descriptive summaries are presented as means and standard deviations for continuous variables and as frequencies for percentages for categorical variables. In addition, study variables were analyzed for distributional properties. The alpha level for significant differences was set at 0.05.

To assess gender differences, an independent-samples t-test was used for continuous variables, a chi-square test for categorical variables, and Fischer’s Exact Test was used if any cell has an expected count less than five. To compare differences between two means, Cohen’s *d*, an effects size, was calculated.

## Results

### Participants’ characteristics

Participants’ characteristics are summarized in Table 1. The gender split was relatively equal, with 46% females and 54% males.

**Table 1:** Demographic characteristics of participants.

Variables	Female	Male	Total
Number (%)	53 (46)	62 (54)	115
Mean age (SD)	24.1 (3.7)	24.1 (3.1)	24.1 (3.4)
Height in cm (SD)	167.3 (6.0)	183.2 (6.7)	176.0 (10.2)
Weight in kg (SD)	70.9 (14.1)	80.0 (11.3)	75.8 (13.4)
Body mass index in kg/m^2^ (SD)	24.7 (5.7)	23.9 (3.3)	24.2 (4.5)
Icelandic as native language n (%)	51 (96.2)	60 (96.8)	111 (96.5)
Mothers’ education level			
Finished university degree n (%)	28 (52.8)	34 (54.8)	62 (54.3)
Finished primary education or less n (%)	7 (13.2)	4 (6.5)	11 (9.5)
Fathers’ education level			
Finished university degree n (%)	20 (37.7)	28 (45.2)	48 (41.4)
Finished primary education or less n (%)	6 (11.3)	5 (8.1)	11 (9.5)
Number of semesters finished at the university			
None- is at my first n (%)	6 (11.3)	7 (11.3)	13 (11.2)
One semester finished is at my second n (%)	45 (84.9)	55 (88.7)	100 (86.9)
Two semesters finished, is at my third n (%)	2 (3.8)	0	2 (1.7)
Marital status			
Single n (%)	24 (45.3)	34 (54.8)	58 (50.0)
In a relationship n (%)	27 (50.9)	27 (43.5)	54 (47.5)
Married n (%)	2 (3.8)	1 (1.6)	3 (2.6)
Work with school n (%)	45 (84.9)	52 (83.9)	97 (83.6)
Quarantine for 3 days or more due to COVID-19 n (%)	18 (34.0)	22 (35.5)	40 (34.8)
Isolation due to COVID-19 n (%)	0	2 (3.2)	2 (0.02)

The average age of participants was 24.1 ± 3.4 years, with a BMI of 24.2 ± 4.5 kg/m^2^. Majority or 96.5% of participants reported Icelandic as their native language, and 83.6% were working along with their studies. In total, 34.8% of participants reported being in quarantine for at least three days or more due to COVID-19, but only two participants (0.02%) had been in isolation. No differences were found in fathers’ or mothers’ educational attainment by participants’ gender.

### Mental health

**Fig 2:**
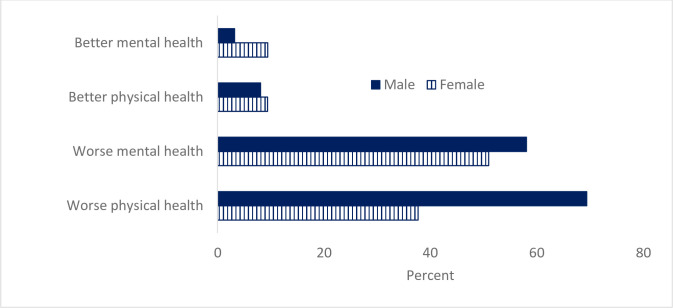
Estimation of mental and physical health compared to health before COVID-19, gender difference found for physical health.

No gender differences were found in participants’ estimation of their mental health state compared to before COVID-19. About 51% of females and 58% males reported their mental health to be worse than before COVID-19, and 9% females and 3% males estimated their mental health to be better than before COVID-19 (Fig 2).

Females had more symptoms of anxiety, *t*(113) = 2.9, p< 0.01, and depression, *t*(113), p<0.05, and less self-esteem, *t*(113), p<0.01, compared to males. The largest effect size was for differences in anxiety. No gender difference was found for body image (Table 2). No difference was found in participants’ mental health by their parents’ level of education.

**Table 2:** Differences in mental health by gender.

	Female	Male	Total	t-value	Cohen’s *d*
Anxiety	7.9 (5.3)	5.3 (4.3)	6.5 (4.9)	**2.9** *	0.55
Body image	11.4 (2.8)	12.3 (2.3)	11.9 (2.5)	1.9	
Depression	23.0 (9.0)	19.8 (5.4)	21.3 (7.4)	**2.4** **	0.44
Self-esteem	29.7 (5.9)	32.7 (4.3)	31.3 (5.3)	**3.1** *	0.51

*p<0.01

**p<0.05

A gender difference was found in participants who experienced stress during COVID-19 compared to before the pandemic, *χ*^*2*^ (1, 115) = 4.5, p = .035. A larger proportion of females, 68%, than males, 48%, experienced more stress than before COVID-19. About the same proportion of females (56%) and males (53%) experienced more loneliness than before COVID-19.

### Perceived health, physical activity, sedentary behavior, and sleep quality

A gender difference was found in participants’ estimation of their physical health (Fisher’s Exact Test p < 0.05). A larger proportion of males, 69%, compared to females, 38%, estimated that their physical health to be worse than before COVID-19, 8% of males and 9% of females estimated it to be better, and 53% of females and 23% of male reported no differences in physical health compared to before COVID-19. No gender difference was found in participants’ BMI (Table 1). No difference was found in participants’ BMI by their parents’ level of education.

A larger proportion of males, 75.8%, than females, 56.6%, estimate their PA to be less than before COVID-19, 15.1% of females and 9.7% of males estimated increase in PA. Regarding sedentary behavior, 71% of females and males estimated it to be more than before the pandemic, and 6% reported it to be less than before COVID-19. About the same percentage, 44%, of females and males estimated their sleep quality worse than before the pandemic, whereas 52% females and 48% males estimated the quality of their sleep the same as before COVID-19.

## Discussion

The main findings of this study were that university students reported a decline in both mental and physical health, their sedentary behavior had increased, and they experienced more loneliness and stress than before the pandemic. In addition, gender differences were found in participants’ estimation of their stress level and physical health during COVID-19 compared to before the start of the pandemic.

### Mental health

The largest effects size found in the difference of mental health across gender was for anxiety followed by self-esteem. Current study mirrors the results of former studies where males experienced fewer symptoms of anxiety and depression compared to the females, and males’ self-esteem was also higher. The few studies which have focused on gender differences in health of university students during COVID-19 in Europe have found gender differences. A French study reported worse mental health among females compared to males [15]. A Spanish research revealed more anxiety and loneliness among females compared to males [23]. However, no gender difference was found for anxiety among college students during the pandemic in China [16]. A study among younger students in Canada (14–18 years old) during COVID-19 found higher depressive symptoms and loneliness among females [24]. Our results are in line with Greek study that similarly found that university students had a higher level of anxiety and depression during COVID-19 [25]. However, it is important to keep in mind that although containment in Iceland were less severe than in other countries it still had negative impact on health. It is noticeable that Icelandic university students are older than students in other Nordic or European countries [26]. The mean age of first-year students in this study was 24-year-old, and 47% reported being in a relationship (2.6% married), and 83% of them worked along with school. With higher age comes more responsibility, including financial and familial duties. Therefore, the Icelandic first-year students might have higher anxiety levels due to worries about extenuating circumstances generated by the pandemic.

This study revealed no gender differences in participants’ body image scores, contrary to previous results that have indicated a clear gender difference, showing males being more satisfied with their body image than females [11]. The lack of gender difference revealed in our study could be related to the fact that males reported a decrease in PA, which is known to be negatively related to body image satisfaction [27]. Due to the closing of sports facilities and strict physical distancing, no organized sport was allowed. The lack of opportunity to exercise could have affected males’ perception of their bodies in the current study. A former study revealed that those who participate in organized sport rate their physical and mental condition and body image more positively than those who do not participate in organized sports [28].

University students are a sensitive group and are worthy of attention as peer social interaction is important for university students, and isolation from friends can cause depression and anxiety. Furthermore, the stress which accompanies the uncertainty about the future due to the pandemic, including economic worries and delays in academic activities, are risk factors for developing anxiety and depression. Not to mention the fear of being infected by the virus might lead to greater anxiety of the university students, especially females [29]. Over half of the participants estimated their mental health to be worse and their stress level and loneliness being more during COVID-19 than before. This finding mirrors the increased need for psychological service reported by the University Student Counselling Center and surveys conducted by the Icelandic Directorate of Health [30].

### Physical health

It was striking that 76% of the males reported their PA had decreased, and 69% of them reported their physical health to be worse than before COVID-19. It must be taken into consideration that strict social distancing regulations due to the pandemic were in place during our data collection, leading to the closing of fitness centers and suspension of organized sports. The fact that such a large portion of the males reported decreased PA and worse physical health could be due to the fact that participation numbers in organized sports are higher for males [31]. Therefore, the strict social distancing regulations impacted them more heavily than females when it came to participation in PA. The suspension of organized sports exercise during the pandemic might therefore explain in part the reported decline in the physical and mental condition of university students in this study.

This study indicated that sedentary behavior increased for both genders during COVID-19. The nature of virtual learning certainly must have contributed to this increase. Almost all lectures for university students took place through online learning platforms. This form of learning demands sitting still in front of the computer for many hours a day, whereas in-person learning requires getting up multiple times a day to move between classrooms, buildings, etc., and therefore automatically increases the PA involvement of university students each day. Our study is in line with a systematic review on sedentary behaviors during COVID-19 [32].

In addition to the decline in mental and physical health, almost half of university students in the study reported that their sleep quality had declined since the beginning of the pandemic. Synergistic factors can cause this as increased sedentary behavior, and lack of exercise can affect sleep quality. Research indicates that exercise has a positive effect on sleep quality and duration [33]. Sufficient sleep and regular exercise are pivotal in maintaining health [34]. During data collection, severe restrictions were in place regarding the opportunity to exercise, as explained above. Fitness centers and sport facilities were closed due to the pandemic, leading to limited possibility to exercise. It is plausible that the lack of exercise of the university students affected their sleep since exercise is known to improve sleep [35].

### Strength and limitation

The main strength of the current study is the equal number of males and females participating which allows for a more gender-specific view on participants’ health. The study also gives an important overview of first-year university students’ health status during the pandemic. However, some limitations must be considered. First, this study is based on a self-reported questionnaire where participants were asked to estimate their mental and physical health compared to before COVID-19. Therefore, current mood states could have affected answers. Second, although representative of Icelandic university students, the sample is reasonably small and relatively homogenous, which could limit the generalizability of our findings. Also, the current study might be prone to selection bias since those who participated could have more interest in health behavior compared to non-participants. Third, the current study does not include results on the possible affects a lockdown can have on mental and physical health, since Iceland has never been in a state of lockdown due to COVID-19. Lastly, the cross-sectional design makes it difficult to determine causality. Therefore, it is important to continue investigating the effects of the pandemic on the mental and physical health of university students to follow up on their well-being as these findings reveal health declines with restrictions even though they don´t include lockdowns. Hopefully, this data can contribute to future studies on the effect of the pandemic on young people and bring to light the importance of support and in-person contact for university students after the pandemic. In the future, after the end of the pandemic, it will be interesting to have these same participants report on their health status again to find out if and how it has changed.

## Conclusion

Our findings suggest that COVID-19 has significantly impaired the physical and mental health of both female and male first-year university students. They were more stressed and lonelier than before COVID-19. The pandemic has left many students feeling overwhelmed as they might be falling behind academically, have lost social connections, quit, or reduced their exercise and sport participation. Young adults’ decline in physical and mental health affects not only the individual itself but can become an economic burden for societies. Therefore, the school and health care systems need to assist young students in unwinding these adverse health and lifestyle changes which have accompanied the pandemic.
